# Spin-Hall-effect-modulation skyrmion oscillator

**DOI:** 10.1038/s41598-020-68710-y

**Published:** 2020-07-20

**Authors:** Hyun-Seok Whang, Sug-Bong Choe

**Affiliations:** Department of Physics and Institute of Applied Physics, Seoul National University, Seoul, 08826 Republic of Korea

**Keywords:** Magnetic devices, Electronic and spintronic devices

## Abstract

The electric-current-induced spin torque on local magnetization allows the electric control of magnetization, leading to numerous key concepts of spintronic devices. Utilizing the steady-state spin precession under spin-polarized current, a nanoscale spin-torque oscillator tunable over GHz range is one of those promising concepts. Albeit successful proof of principles to date, the spin-torque oscillators still suffer from issues regarding output power, linewidth and magnetic-field-free operation. Here we propose an entirely new concept of spin-torque oscillator, based on magnetic skyrmion dynamics subject to lateral modulation of the spin-Hall effect (SHE). In the oscillator, a skyrmion circulates around the modulation boundary between opposite SHE-torque regions, since the SHE pushes the skyrmion toward the modulation boundary in both regions. A micromagnetic simulation confirmed such oscillations with frequencies of up to 15 GHz in media composed of synthetic ferrimagnets. This fast and robust SHE-modulation-based skyrmion oscillator is expected to overcome the issues associated with conventional spin-torque oscillators.

## Introduction

Spin-torque oscillators offer promising applications such as wide-range-tunable frequency generation/detection^1,2^, signal processing^3^, and dynamic recording^4^. The spin-torque nano-oscillator (STNO)^5–8^ was the first example, which utilizes the spin precession induced by the spin-polarized current^5,9–12^ passing through the point contact. Despite a successful demonstration of its high oscillation frequency with wide-ranging tunability^5,9–12^, the STNOs still require improvement of their output power and linewidth, as well as their method of magnetic-field-free operation^13^. To overcome these issues, techniques such as synchronization between multiple point contacts^14^ and self-injection locking^15^ have been investigated. Otherwise, an enhanced output power with a much narrower linewidth has been attained by a spin-torque vortex oscillator (STVO)^16,17^, which utilizes the gyration of a vortex core, confined within a point contact. By replacing the vortex core with a magnetic skyrmion, the concept of a spin-torque skyrmion oscillator (STSO)^18–24^ has also been suggested, which would further improve the output power and be able to operate without an external magnetic field.

The narrow linewidth of the STVOs and STSOs is inherently attributed to the larger magnetic volume involved in the gyration^16^. However, the resulting frequencies are limited up to a maximum of a few GHz, which is much lower than typical STNOs^16–20^. To increase the frequencies, it is essential to improve the speed efficiency of the vortex and skyrmion to the external current. Recently, many studies have reported on faster domain walls and skyrmions at the angular-momentum-compensation points of synthetic ferrimagnetic (SFi) systems^25–27^. It has also been shown that the frequency of a STSO can increase up to tens of GHz utilizing the SFi systems^21^. Albeit all these promising qualities, there remains several issues in the STSOs. Due to the current-perpendicular-to-plane (CPP) geometry of the conventional nano-pillar structures^18,21^, it is challenging to separate the detecting current channel^19,20^ from the driving current channel without breaking the cylindrical symmetry. Moreover, the driving channel is relatively large, of which the cross section has to cover the whole area of the skyrmion oscillation path. These issues can be solved by introducing current-in-plane (CIP) geometry, which enables easy separation of the detecting and driving current channels and also, reduces the cross-sectional area of the driving current channel. In this sense, we propose a new spin-torque oscillator—namely, the spin-Hall-effect-modulation skyrmion oscillator (SHEM-SO)—utilizing skyrmion motion directly driven by the horizontal SHE current. In this SHEM-SO geometry, the CPP detecting channel can be separated from the CIP driving channel and also, the cross sectional area of the CIP driving channel is reduced in comparison to the CPP driving channel by the factor of the film thickness (several nanometers) over the skyrmion oscillation path diameter (several tens of nanometers). As the cross-sectional area decreases, the total driving current through the structure and thus, the operation power also decreases by the same factor for the case of a given driving current density. By utilizing a SFi system, we demonstrate that the SHEM-SO produces a high frequency comparable to the STNOs, while maintaining all the other merits including the narrow linewidth of the STVOs.

## Results

**Figure 1:**
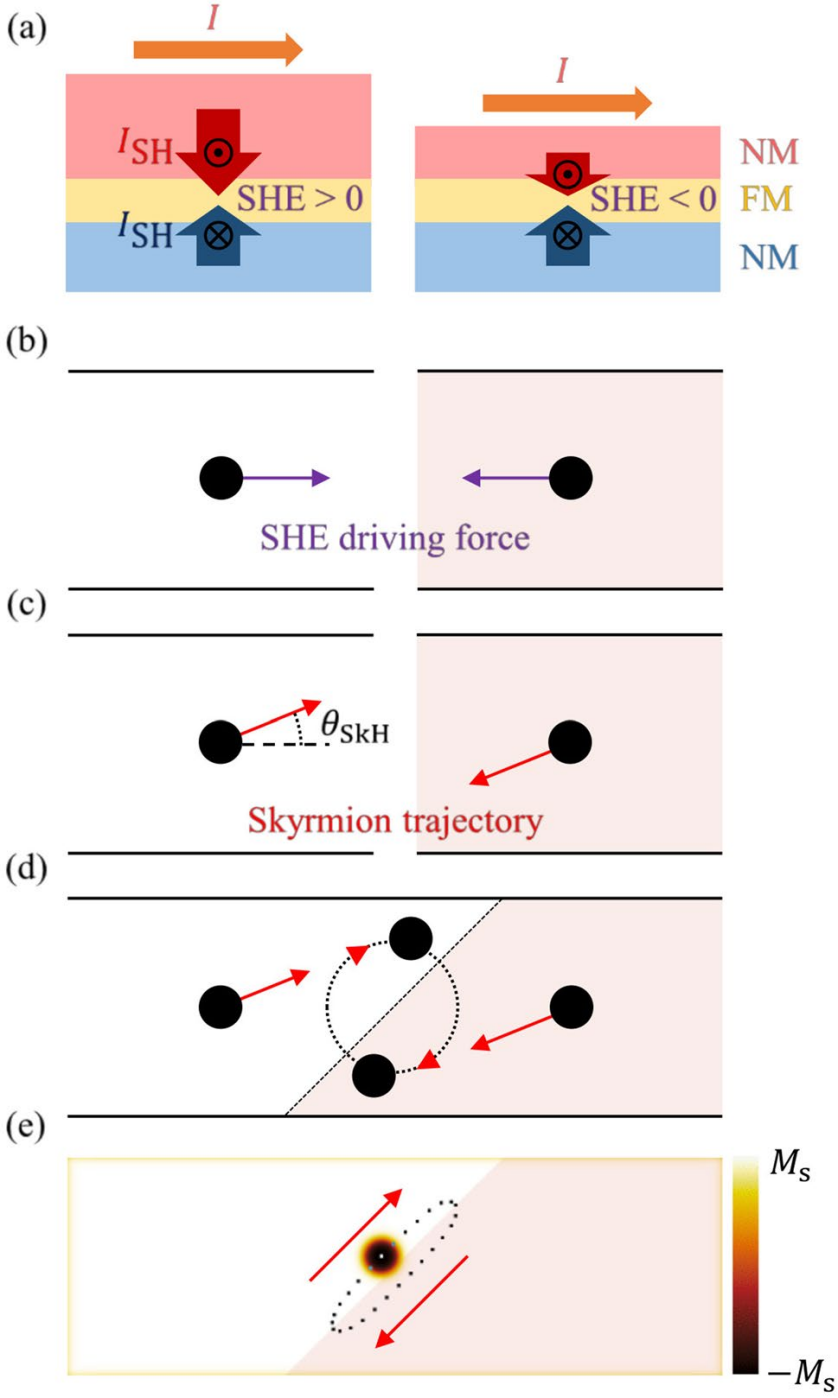
SHEM-SO operation principle. (**a**) Cross-sectional view of layered structure with SHE modulation. The red, yellow, and blue areas indicate the top NM, FM, and bottom NM layers, respectively. The orange arrows show the direction of $I$. The red and blue arrows are the directions of $I_{SH}$ from the top and bottom NM layers, respectively. (**b**) Top view of each layered structure. The white and light-pink areas indicate the regions of the opposite SHE signs. The black circular dots represent skyrmions. The purple arrows show the SHE-induced driving forces. (**c**) The red arrows show the skyrmion trajectories with an angle $\theta_{SkH}$. (**d**) Schematic of closed skyrmion path in conjoined SHE modulation structure. (**e**) Simulation result of steady-state skyrmion oscillation. The color corresponds to the out-of-plane component of the magnetization as indicated by the color bar on the right. The image in (**e**) has been obtained with OOMMF (version 2.0a, https://math.nist.gov/oommf/)^35^.

Figure 1a is a schematic illustration of the spin-Hall current being injected from adjacent nonmagnetic (NM) layers into the ferromagnetic (FM) layer. Upon the injection of an electric current ($I$), the top and bottom NM layers generate vertical spin-Hall current ($I_{SH}$) of opposite spin polarizations^28,29^. The counterbalance between the $I_{SH}$’s of each NM layer determines the net spin polarization injected into the FM layer. Since the amount of the $I_{SH}$’s depends on the thicknesses of the NM layers^29,30^, it is possible to control the sign of the net spin polarization by adjusting the thicknesses of the NM layers, as exemplified by the two tri-layered structures with thicker and thinner top NM layers. These two structures experience opposite signs of net spin polarization and consequently opposing SHE torques (Fig. 1b).

Due to the skyrmion-Hall effect^31,32^, the gyrational torque tilts the skyrmion trajectories by the skyrmion-Hall angle ($\theta_{SkH}$) from the driving force direction (Fig. 1c). Note that these skyrmion trajectories are bound to opposite sides of the wire. Due to these reversed tendencies to the wire sides, if these two structures are joined as shown in Fig. 1d, it becomes possible for a skyrmion to form a closed oscillation path around the modulation boundary. We named this system “SHEM-SO”.

A micromagnetic simulation was performed to confirm this prediction (see “Methods” section for parameter details). The skyrmion indeed exhibited closed steady-state oscillation around the modulation boundary (Fig. 1e) (see also Supplementary Movie S1, 2). The simulation also confirmed that a skyrmion converges to a single steady-state oscillation path regardless of its initial position. Since the oscillation continued for more than thousands of periods (~ 10,000 ns), we could conclude that the oscillation is not a transient behavior.

**Figure 2:**
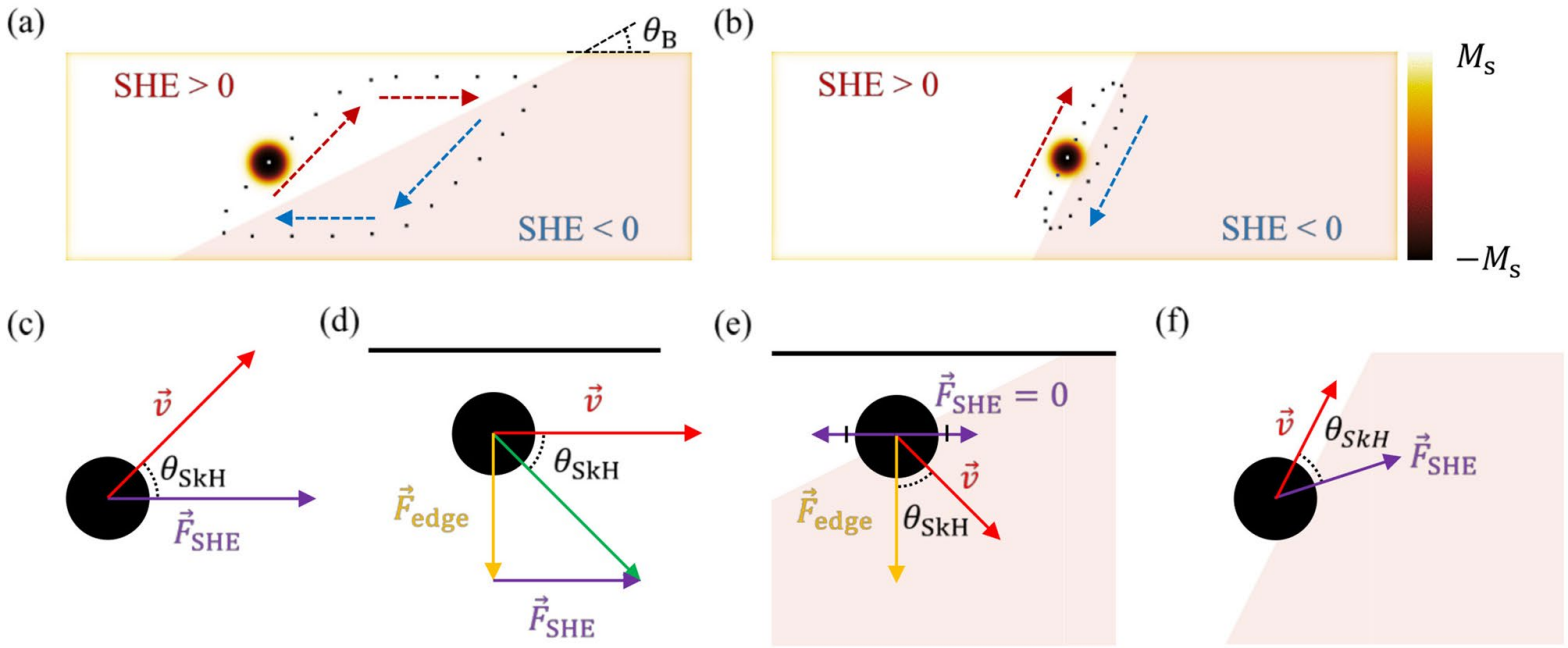
Simulated skyrmion paths and schematics of skyrmion dynamics in each process. Simulated results for skyrmion oscillation paths for (**a**) $\theta_{SkH} > \theta_{B}$ and (**b**) $\theta_{SkH} < \theta_{B}$. The white and light-pink areas indicate the regions of the opposite SHE signs, modulated by an angle $\theta_{B}$. Schematics of skyrmion dynamics for the areas: (**c**) far away from the modulation boundary, (**d**) near an edge, (**e**) near an edge and modulation boundary, and (**f**) along the modulation boundary. The purple, yellow, and red arrows indicate the directions of the $\vec{F}_{SHE}$, $\vec{F}_{Edge}$, and skyrmion velocity ($\vec{v}$). The black horizontal lines in (**d**) and (**e**) represent the upper edge of the wire. The image in (**a**) and (**b**) has been obtained with OOMMF (version 2.0a, https://math.nist.gov/oommf/)^35^.

The key features of the SHEM-SO are the non-zero $\theta_{SkH}$ and the tilted modulation boundary with an angle ($\theta_{B}$). Depending on the relation between $\theta_{SkH}$ and $\theta_{B}$, two distinctive oscillation paths appear. Figure 2 depicts (a) the parallelogram-like path for $\theta_{SkH} \ge \theta_{B}$ and (b) the parallel path along the modulation boundary for $\theta_{SkH} < \theta_{B}$, respectively.

For $\theta_{SkH} \ge \theta_{B}$, the SHE torque exerts a driving force on the skyrmion in the direction of the electric current and then, the skyrmion moves in the direction at an angle $\theta_{SkH}$ from the $\vec{F}_{SHE}$ (Fig. 2c). As the skyrmion approaches the edge, the edge repulsion force ($\vec{F}_{edge}$) increases until the skyrmion moves parallel to the edge (Fig. 2d);The $\vec{F}_{edge}$ comes from the exchange interaction between the opposite in-plane magnetizations of the wire edge and the skyrmion boundary, whose magnetization directions are fixed due to the chirality induced by the Dzyaloshinskii–Moriya interaction (DMI). When the skyrmion reaches the modulation boundary, the $\vec{F}_{SHE}$ starts to decrease due to the cancellation between the opposite SHE regions and the skyrmion drifts away from the edge by $\vec{F}_{edge}$ (Fig. 2e). The same processes then repeat in the opposite SHE region to form a closed path.

For $\theta_{SkH} < \theta_{B}$, the skyrmion first reaches the modulation boundary. The net $\vec{F}_{SHE}$ at the modulation boundary now tilts away from the electric current direction until the trajectory becomes parallel to the modulation boundary (Fig. 2f) (see Supplementary Discussion). Then, upon approaching the wire edge, $\vec{F}_{edge}$ pushes the skyrmion into the opposite SHE region (Fig. 2e). The same processes then repeat in the opposite SHE region to form a closed path.

**Figure 3:**
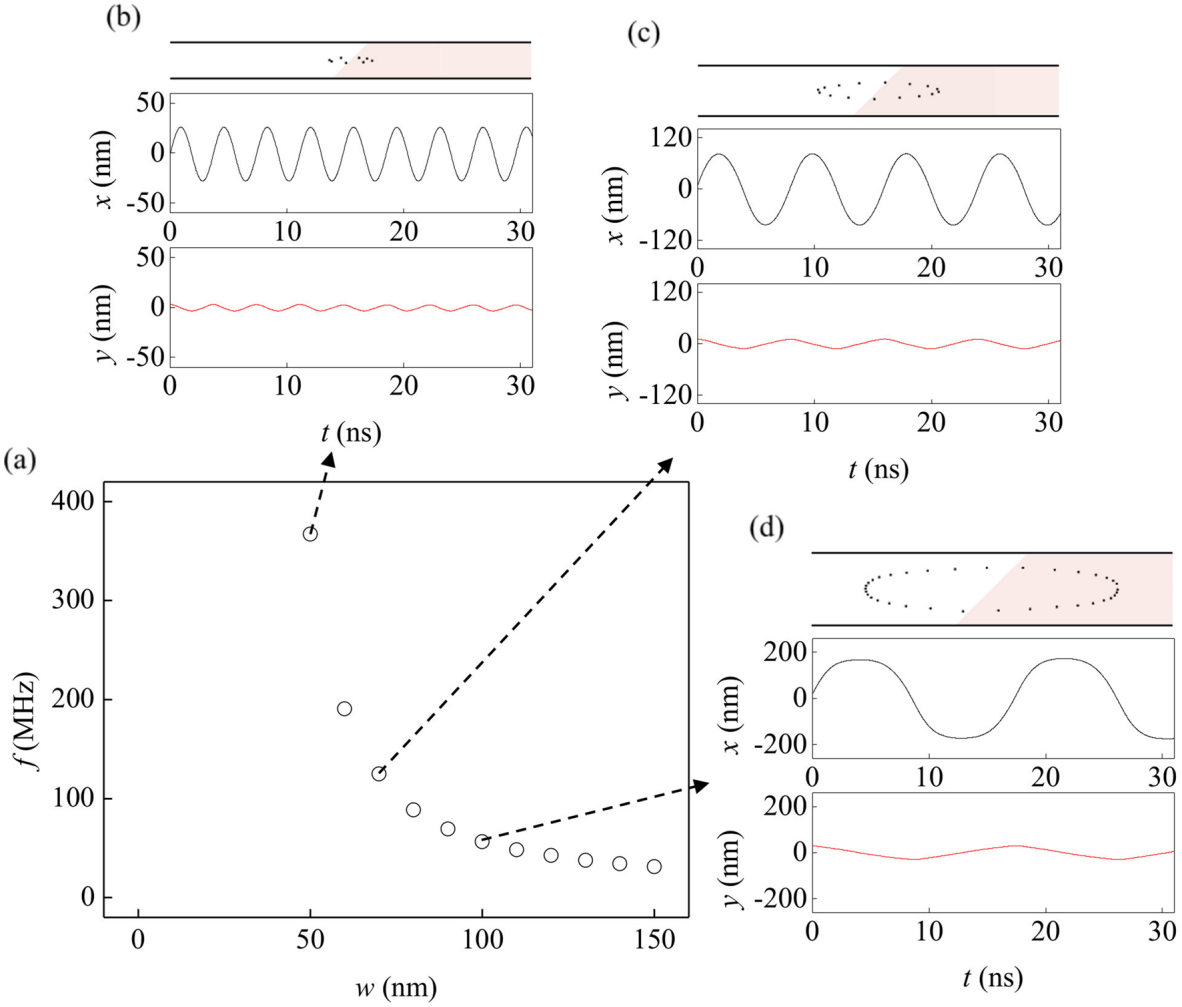
Oscillation frequency and paths with respect to wire width. (**a**) Plot of $f$ with respect to $w$. (**b**–**d**) Oscillation paths and plot of the $x$ and $y$ positions with respect to time $t$ for wires for which $w =$50, 70, 100 nm, respectively. The image in (**b**), (**c**) and (**d**) has been obtained with OOMMF (version 2.0a, https://math.nist.gov/oommf/)^35^.

The oscillation characteristics of the SHEM-SO is now discussed. The length of the oscillation path and the skyrmion speed along the path determine the oscillation frequency ($f$). For heuristic reasons, $\theta_{B}$ is fixed to 45°, since $f$ is a slow varying function of $\theta_{B}$ with a maximum near $\theta_{B} \cong$ 45° (see Supplementary Discussion). Since the oscillation path is confined within the wire, the path length directly depends on the wire width ($w$) and therefore, a narrower wire is preferred for a higher $f$. The simulation results confirm that $f$ is inversely proportional to $w$ over a range down to the practical lower limit (around 50 nm), close to the skyrmion size (Fig. 3a).

For fixed oscillation paths with a given geometry of $w$ and $\theta_{B}$, the skyrmion speed now solely determines $f$. Since the skyrmion motion is driven by the SHE torque by a DC current, a higher current density ($J$) leads to a faster skyrmion and consequently, a higher $f$. However, since $J$ also increases the skyrmion-Hall force, the skyrmions can be destroyed at the wire edge, if one injects excessively large $J$. Therefore, the maximum applicable current density ($J_{max}$) is determined by the counterbalance between the skyrmion-Hall force and the edge-repulsion force. The maximum possible frequency ($f_{max}$) is attained at $J_{max}$.

**Figure 4:**
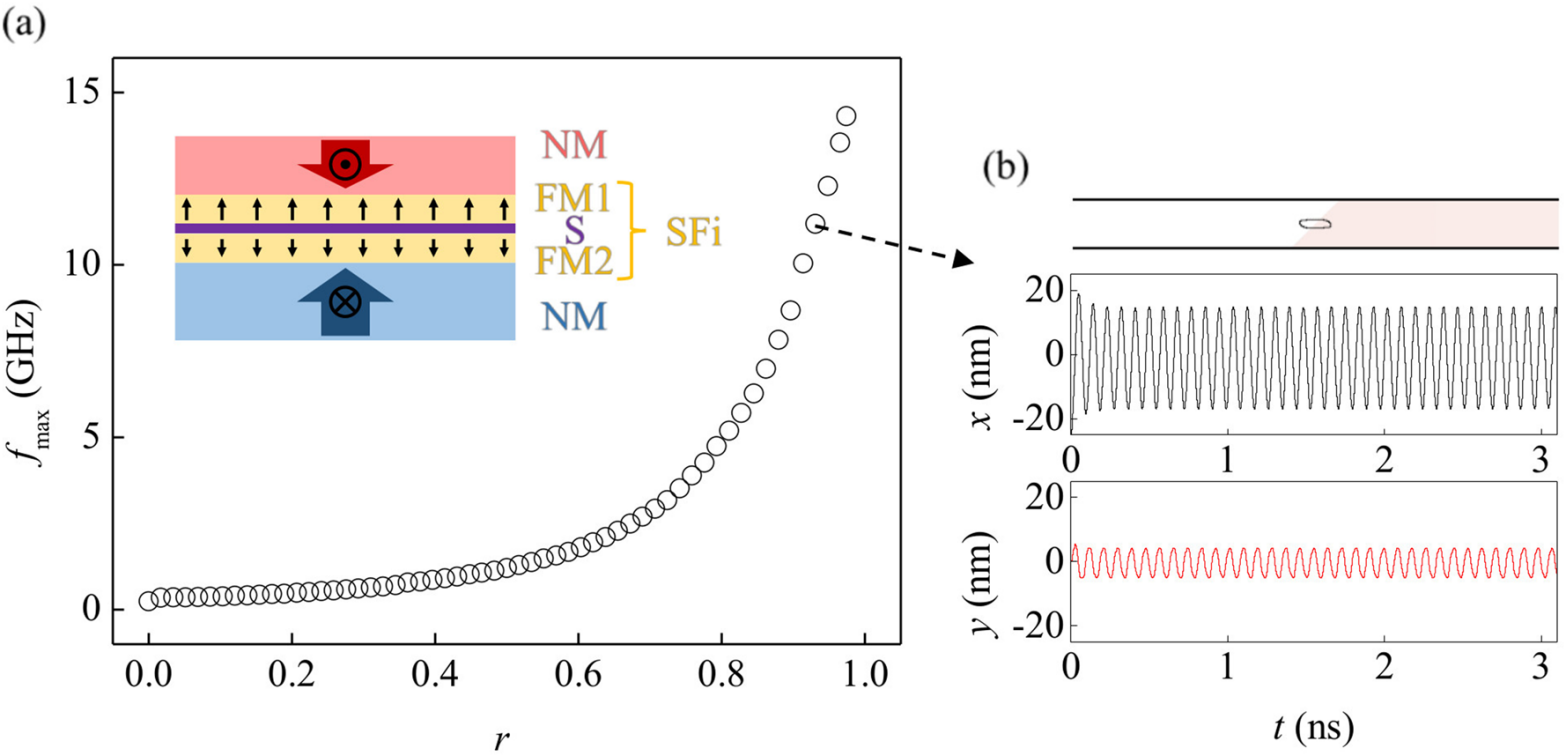
Oscillation frequency with respect to angular momentum ratio of synthetic ferrimagnet. (**a**) Plot of $f_{max}$ with respect to $r$. Inset illustrates the synthetic ferrimagnetic multilayer structure composed of two ferromagnetic layers (FM1 and FM2) and a spacer (S). (**b**) Oscillation paths and plot of $x$ and $y$ positions with respect to time $t$ for $r =$ 0.93. The image in (**b**) has been obtained with OOMMF (version 2.0a, https://math.nist.gov/oommf/)^35^.

To increase $f_{max}$ further, the SFi system was adopted that are known to exhibit high DW/skyrmion speeds near the angular-momentum compensation point^25–27^. The SFi system consists of two FM layers with an antiferromagnetic coupling separated by a spacer. By controlling the compositions of the FM layers, the ratio $r$ ($\equiv \gamma_{2} M_{S1} t_{1} /\gamma_{1} M_{S2} t_{2}$) can be adjusted to reach the angular-momentum compensation condition (i.e. $r = 1$), where $\gamma_{1,2}$, $M_{S1,2}$, and $t_{1,2}$ are the gyromagnetic constants, saturation magnetizations, and thicknesses of the FM layers, respectively. As $r$ approaches 1, the SHE-torque efficiency increases considerably and the gyroscopic force eventually decreases to zero^25–27^. The former enhances the skyrmion speed while the latter enhances $J_{max}$. Figure 4 clearly shows that $f_{max}$ increases drastically as $r$ approaches 1 and, $f_{max}$ up to about 15 GHz is successfully demonstrated at $J_{max} = 5.0 \times 10^{11} \;{\text{A/m}}^{2}$, which corresponds to 30 μA through the magnetic layer.

## Discussion

Ideally, the SHEM-SO can exhibit frequencies from 0 to $f_{max}$ since the magnitude of the applied dc current can linearly control the speed of a skyrmion and the resulting frequencies of the system. Thus, highly tunable nanoscale oscillator can be realized via the SHEM-SO. Additionally, one can find in the Fig. 3b–d, that as $w$ decreases, the skyrmion motion becomes more confined, but still exhibits an elliptical path, elongated along the wire length. Since the skyrmion is driven by the CIP geometry, it is easy to put a CPP magnetic tunnel junction (MTJ) nano-pillar structure on the tip of the elliptical path and thus, makes it possible to detect a large output signal. Since the magnetization under nano-pillar completely switches in this case unlike the STNOs, where the magnetization rotates slightly away from the magnetization direction of the pinned layer, the output power from magnetoresistance (MR) effect is expected to increase significantly. Moreover, a skyrmion-creation channel can be placed outside the oscillation area, where the created skyrmion can be transported to the oscillation area. Therefore, the oscillation, detection, and creation can be operated independently by different channels, allowing much more versatile architecture. Alike the STVOs where the oscillation of a vortex core exhibits narrow linewidth^16^, the large magnetic volume of the well-defined skyrmion structure is also expected to provide a robust oscillation resulting in a narrow linewidth. Since the SHEM-SO operation does not require external magnetic field, the SHEM-SO can solve all the issues of the conventional spin-torque oscillators, with frequencies up to tens of GHz utilizing the SFi systems.

Although we can claim that the SHEM-SO can have all the great features described above, we should also discuss the obstacles in realizing the concept. First, we assume that a stable and isolated skyrmion that does not annihilate at the wire edge is possible, while this is not true at least for now. Further studies to realize a stable skyrmion is needed. Second, the SHEM-SO does not account for the nucleation of a skyrmion, which is a quest of its own. Although we stated that a separate nucleation channel can be placed outside the oscillation area, the nucleation method should be well established beforehand. Most nucleation methods include perturbation of a skyrmion-favoring system, mostly with high enough current^33,34^. The method described in the Ref.^34^ should be attachable to the SHEM-SO rather easily, since it is also in CIP geometry. Finally, for a reading mechanism, integrating a small MTJ onto the oscillating path of a skyrmion would not be so easy since the frequency of the SHEM-SO is directly related to the dimension. Scaling an MTJ would be a bottleneck in realizing high-frequency SHEM-SO.

In summary, we have proposed an entirely new concept of a spin-torque oscillator. With the promising features regarding output power, linewidth, magnetic-field-free operation and versatile CIP geometry, the SHEM-SO can open a whole new chapter of designing nanoscale tunable microwave oscillators. Although the concept still faces some obstacles to overcome, such as the realization of a stable skyrmion and integrating/scaling rather complicated structure, the realization of the SHEM-SO would not be too hard/far considering all the efforts being put into skyrmion studies nowadays.

## Methods

### Micromagnetic simulation

A finite-difference micromagnetic simulation was carried out using OOMMF code^35^ with a DMI module^36^. The cell-size was set to 1 nm * 1 nm * x (thickness of a single ferromagnetic layer) nm. For the tri-layered ferromagnetic films (Figs. 1, 2, 3), the FM layer thickness was set to 0.6 nm. Typical magnetic parameters of Pt/Co/Pt films were used^33^, that are 580 kA/m for the saturation magnetization, 15 pJ/m for the exchange stiffness (~ 8.4 nm exchange length), 0.8 mJ/m^3^ for the perpendicular magnetic anisotropy, and 3.5 mJ/m^2^ for the DMI strength. For the synthetic ferromagnetic films (Fig. 4), the thicknesses of the two FM and space layers were set to 0.4 nm. The same magnetic parameters as those of the above tri-layered ferromagnetic films were used, with the exception of the magnetization of the top FM2 layer, which varied from 0 to 580 kA/m. The exchange stiffness between the layer^26^ was set to − 0.3 pJ/m. The damping-like spin-orbit torque efficiency from the SHE was set to ± 10^–13^ Tm^2^/A for each opposite SHE region. The current density varied over a range of 0.01–0.5 × 10^12^ A/m. The Gilbert damping parameter was 0.01 in most cases, except in the case of Figs. 1 and 2 that utilized a value large enough to ensure the clear visualization of the oscillation paths. Finally, the initial state of the magnetization was manually set to be an isolated single skyrmion, which automatically stabilizes into its energetically-favored profile and size (see Supplementary Discussion for the image of the initial state). The type of a skyrmion was Nèel-type and the chirality were chosen to be counter clockwise, however the chirality does not affect the result in any ways, since it only reverts the direction of an oscillation.

## Supplementary information

Supplementary Information 1.

Supplementary Information 2.

Supplementary Video 1.

Supplementary Video 2.
