# Supplementary material for: Spin-Hall-effect-modulation skyrmion oscillator
Source: Sci Rep. 2020 Jul 20;10:11977. doi: 10.1038/s41598-020-68710-y (PMC7371710; doi:10.1038/s41598-020-68710-y)
Supplement: Supplementary file 1 — Supplementary Information 1. [file 41598_2020_68710_MOESM1_ESM.docx]

Supplementary Information Guide for

**Spin-Hall-Effect-Modulation Skyrmion Oscillator**

Hyun-Seok Whang, Sug-Bong Choe^*^

Correspondence to: [sugbong@snu.ac.kr](mailto:sugbong@snu.ac.kr)

**Titles and text summaries of Supplementary Files**

“Supplementary Information.pdf”: **Supplementary Discussion**: 3 display items regarding the Supplementary Discussion are contained. The text discusses the initial magnetization state of the simulation, the analytic formula explaining the mechanism of SHEM**-**SO, the modification of the formula for synthetic ferrimagnets and the angle dependence of the frequency of SHEM**-**SO.

“Supplementary Video S1.mp4”: **Skyrmion Oscillation type 1**: Simulated result of the skyrmion oscillation in the configuration of Fig. 2a in the main text. 1 second in the movie corresponds to 3 ns in the simulation time.

“Supplementary Video S2.mp4”: **Skyrmion Oscillation type 2**: Simulated result of the skyrmion oscillation in the configuration of Fig. 2b in the main text. 1 second in the movie corresponds to 3 ns in the simulation time.
