# Supplementary material for: Spin-Hall-effect-modulation skyrmion oscillator
Source: Sci Rep. 2020 Jul 20;10:11977. doi: 10.1038/s41598-020-68710-y (PMC7371710; doi:10.1038/s41598-020-68710-y)
Supplement: Supplementary file 2 — Supplementary Information 2. [file 41598_2020_68710_MOESM2_ESM.docx]

Supplementary Materials for

**Spin-Hall-Effect-Modulation Skyrmion Oscillator**

Hyun-Seok Whang, Sug-Bong Choe^*^

Correspondence to: [sugbong@snu.ac.kr](mailto:sugbong@snu.ac.kr)

**Contents:**

Supplementary Discussion

Figures. S1 to S3

**Supplementary Discussion**

Initial state of a skyrmion

A Nèel-type skyrmion can be placed anywhere since the skyrmion eventually moves toward the modulation boundary where the same oscillation happens. The initial magnetic profile is shown in the Fig. S1.

Thiele formula for skyrmion motion near modulation boundary

The simulation is based on the Landau-Lifshitz-Gilbert equation with a damping-like SHE torque term.

$\frac{\partial\vec{m}}{\partial t}=-\gamma\vec{m}\times H+\alpha\vec{m}\times\frac{\partial\vec{m}}{\partial t}+\gamma\epsilon_{\mathrm{SHE}}\vec{m}\times(\vec{m}_{p}\times\vec{m})$. (1)

When a Nèel-type skyrmion with cylindrical symmetry is driven by a driving force $\left( F_{x}^{\mathrm{ext}},F_{y}^{\mathrm{ext}} \right)$, the steady-state velocity $\left( v_{x},v_{y} \right)$ of the skyrmion is given by

$\left( \begin{matrix} v_{x} \\ v_{y} \end{matrix} \right)=\frac{1}{{(\alpha D)}^{2}+G^{2}}\left( \begin{matrix} \alpha D & G \\ -G & \alpha D \end{matrix} \right)\left( \begin{matrix} F_{x}^{\mathrm{ext}} \\ F_{y}^{\mathrm{ext}} \end{matrix} \right)$, (2)

based on the Thiele formula of the Landau-Lifshitz-Gilbert equation (1), where $\alpha$ is the damping parameter, $G$ is the integrated gyration term, and $D$ is the integrated dissipation term. By defining the skyrmion Hall angle $\theta_{\mathrm{SkH}}$ as $\tan\theta_{\mathrm{SkH}}=-\frac{G}{\alpha D}$, the equation can be rewritten as

$\left( \begin{matrix} v_{x} \\ v_{y} \end{matrix} \right)=\frac{1}{\sqrt{{(\alpha D)}^{2}+G^{2}}}\left( \begin{matrix} \cos\theta_{\mathrm{SkH}} & -sin \theta_{\mathrm{SkH}} \\ \sin\theta_{\mathrm{SkH}} & \cos\theta_{\mathrm{SkH}} \end{matrix} \right)\left( \begin{matrix} F_{x}^{\mathrm{ext}} \\ F_{y}^{\mathrm{ext}} \end{matrix} \right)$. (3)

Eq. (2) indicates that a skyrmion moves in a direction with a fixed angle of $\theta_{\mathrm{SkH}}$ from the external driving force, as discussed in the main text and Fig. 2. The SHE-induced driving force under the injection of a current density $J$ is then given by the integration over the entire film area $A$ as

$F_{i}^{\mathrm{SHE}}=-\gamma\epsilon_{\mathrm{SHE}}J\int\vec{m}_{p}\cdot\left( \frac{\partial\vec{m}}{\partial x_{i}}\times\vec{m} \right)dA$, (4)

where $\gamma$ is the gyromagnetic ratio and $\epsilon_{\mathrm{SHE}}$ is the SHE efficiency. Here, $\vec{m}$ is the unit vector of the magnetization in the FM layer and $\vec{m}_{p}$ is the unit vector of the spin polarization of the SHE electrons injected from the NM layers. In this formula, $\vec{m}_{p}$ is perpendicular to the direction of $J$ within the film plane.

To estimate $F_{i}^{\mathrm{SHE}}$ across the modulation boundary, we assume rigid skyrmions composed of a Nèel-type domain wall with cylindrical symmetry and then, with some rewriting, the skyrmion texture can be expressed as a cylindrical coordinate $\left( \rho,\varphi,z \right)$ as

$\left( \begin{matrix} m_{\rho} \\ m_{\varphi} \\ m_{z} \end{matrix} \right)=\frac{N}{\cosh^{2} \left( \frac{l}{\lambda} \right)+\sinh^{2} \left( \frac{\rho}{\lambda} \right)}\left( \begin{matrix} 2d\cosh\left( \frac{l}{\lambda} \right)\sinh\left( \frac{\rho}{\lambda} \right) \\ 0 \\ \cosh^{2} \left( \frac{l}{\lambda} \right)-\sinh^{2} \left( \frac{\rho}{\lambda} \right) \end{matrix} \right)$, (5)

or as a Cartesian coordinate $\left( x,y,z \right)$ as

$\left( \begin{matrix} m_{x} \\ m_{y} \\ m_{z} \end{matrix} \right)=\frac{N}{\cosh^{2} \left( \frac{l}{\lambda} \right)+\sinh^{2} \left( \frac{\sqrt{x^{2}+y^{2}}}{\lambda} \right)}\left( \begin{matrix} \frac{x}{\sqrt{x^{2}+y^{2}}}\left[ 2d\cosh\left( \frac{l}{\lambda} \right)\sinh\left( \frac{\sqrt{x^{2}+y^{2}}}{\lambda} \right) \right] \\ \frac{y}{\sqrt{x^{2}+y^{2}}}\left[ 2d\cosh\left( \frac{l}{\lambda} \right)\sinh\left( \frac{\sqrt{x^{2}+y^{2}}}{\lambda} \right) \right] \\ \cosh^{2} \left( \frac{l}{\lambda} \right)-\sinh^{2} \left( \frac{\sqrt{x^{2}+y^{2}}}{\lambda} \right) \end{matrix} \right)$, (6)

where $l$ is the skyrmion size and $\lambda$ is the domain wall width. Here, $d$ is the sign of the DMI and $N$ is the topology number (= ±1) depending on the magnetic polarity inside the skyrmion. By applying the skyrmion texture defined by Eq. (5), the integration in Eq. (4) can be rewritten as

$\tilde{F}_{x,y}^{\mathrm{SHE}}=-\gamma\epsilon_{\mathrm{SHE}}J\int_{-\infty}^{\infty} \int_{-\infty}^{\infty} f_{x,y}\left( x,y \right)dxdy$, (7)

where the force densities $f_{x}\left( x,y \right)$ and $f_{y}\left( x,y \right)$ are defined by

$\left\{ \begin{matrix} \begin{matrix} f_{x}\left( x,y \right)= & 8\cosh\left( \frac{l}{\lambda} \right)/\left\{ \left( x^{2}+y^{2} \right)\left[ \sinh^{2} \left( \frac{\sqrt{x^{2}+y^{2}}}{\lambda} \right)+\cosh^{2} \left( \frac{l}{\lambda} \right) \right]^{2} \right\} \\ & \times\left\{ \sinh^{2} \left( \frac{\sqrt{x^{2}+y^{2}}}{\lambda} \right)\left[ x^{2}\cosh\left( \frac{\rho}{\lambda} \right)-\frac{l}{\sqrt{x^{2}+y^{2}}}y^{2}\sinh\left( \frac{\sqrt{x^{2}+y^{2}}}{\lambda} \right) \right] \right. \\ & \text{ }\left. +\cosh^{2} \left( \frac{l}{\lambda} \right)\left[ x^{2}\cosh\left( \frac{\sqrt{x^{2}+y^{2}}}{\lambda} \right)+\frac{l}{\sqrt{x^{2}+y^{2}}}y^{2}\sinh\left( \frac{\sqrt{x^{2}+y^{2}}}{\lambda} \right) \right] \right\} \end{matrix} \\ \begin{matrix} f_{y}\left( x,y \right)= & 2xy\cosh\left( \frac{l}{\lambda} \right)/\left\{ \left( x^{2}+y^{2} \right)\left[ \sinh^{2} \left( \frac{\sqrt{x^{2}+y^{2}}}{\lambda} \right)+\cosh^{2} \left( \frac{l}{\lambda} \right) \right]^{2} \right\} \\ & \times\left\{ \cosh^{2} \left( \frac{l}{\lambda} \right)\left[ \cosh\left( \frac{\sqrt{x^{2}+y^{2}}}{\lambda} \right)-\frac{l}{\sqrt{x^{2}+y^{2}}}\sinh\left( \frac{\sqrt{x^{2}+y^{2}}}{\lambda} \right) \right] \right. \\ & \text{ }\left. +\sinh^{2} \left( \frac{\sqrt{x^{2}+y^{2}}}{\lambda} \right)\left[ \cosh\left( \frac{\sqrt{x^{2}+y^{2}}}{\lambda} \right)+\frac{l}{\sqrt{x^{2}+y^{2}}}\sinh\left( \frac{\sqrt{x^{2}+y^{2}}}{\lambda} \right) \right] \right\} \end{matrix} \end{matrix} \right.$, (8)

in the case that $J$ is injected along the $x$ axis and thus, $\vec{m}_{p}$ is parallel to the $y$ axis. Inside an area of uniform SHE, the integration of Eq. (7) gives $\tilde{F}_{y}^{\mathrm{SHE}}=0$ and thus, the SHE-induced driving force is parallel to the current direction in the uniform SHE region.

When a skyrmion approaches the modulation boundary by a normal distance $\delta$, Eq. (6) is modified to

$\begin{matrix} F_{x,y}^{\mathrm{SHE}}\left( \delta\right)= & -\gamma\epsilon_{\mathrm{SHE}}J\left( \int_{-\infty}^{\infty} \int_{-\infty}^{x\tan\theta_{\text{B}}} f_{x,y}\left( x-\delta\sin\theta_{\text{B}}, y+\delta\cos\theta_{\text{B}} \right)dxdy \right. \\ & \left. \text{ }-\int_{-\infty}^{\infty} \int_{x\tan\theta_{\text{B}}}^{\infty} f_{x,y}\left( x-\delta\sin\theta_{\text{B}}, y+\delta\cos\theta_{\text{B}} \right)dxdy \right) \end{matrix}$, (9)

where $\theta_{\text{B}}$ is the angle of the modulation boundary. Note that $\tilde{F}_{x,y}^{\mathrm{SHE}}$ in Eq. (7) equals $F_{x,y}^{\mathrm{SHE}}\left( -\infty\right)$ in Eq. (9). Figure S2a plots the numerical calculation results of $F_{x,y}^{\mathrm{SHE}}$. The plots clearly show that $F_{x}^{\mathrm{SHE}}$ monotonically decreases to zero with $\delta$. On the other hand, $F_{y}^{\mathrm{SHE}}$ increases from zero to a maximum and then vanishes as $\delta$ approaches to zero. Then, as shown in Fig. S2b, the angle $\theta_{\text{SHE}}$ ($=\tan^{-1} \left( F_{y}^{\mathrm{SHE}}/F_{x}^{\mathrm{SHE}} \right)$) increases as $\delta$ decreases and eventually, converges to a certain angle. When $\theta_{\text{SHE}}$ becomes an angle $\theta_{\text{B}}-\theta_{\text{SkH}}$, the skyrmion moves parallel to the modulation boundary as described in Fig. 2f.

Thiele formula for synthetic ferrimagnets

For synthetic ferromagnetic films, $G$ is rewritten as

$G=4\pi N\frac{\left( {\gamma_{2}M}_{s1}t_{1}-\gamma_{1}M_{s2}t_{2} \right)}{\left( \gamma_{2}M_{s1}t_{1}+\gamma_{1}M_{s2}t_{2} \right)}$, (10)

when synthetic ferrimagnetic films are composed of two FM layers with a gyromagnetic ratio $\gamma_{1,2}$, saturation magnetizations $M_{s1,2}$, and thicknesses $t_{1,2}$ for each FM layer, respectively. Since the steady-state skyrmion speed is proportional to $\frac{1}{\sqrt{{(\alpha D)}^{2}+G^{2}}}$, as given by Eq. (3), the skyrmion speed increases as $G$ decreases and approaches zero when $\gamma_{2}M_{s1}t_{1}\cong\gamma_{1}M_{s2}t_{2}$ near the angular-momentum compensation condition. Under this condition, $\theta_{\mathrm{SkH}}$ decreases to zero through the relation $\tan\theta_{\mathrm{SkH}}\propto G$ and therefore, $J_{\text{max}}$ increases with a reduction in the skyrmion-Hall-effect-induced gyroscopic force counterbalancing the edge repulsion force. Combining these two effects, the maximum skyrmion speed and, thereby, $f_{\text{max}}$, increases in the synthetic ferromagnetic films near the angular-momentum compensation condition.

Frequency variation with respect to the angle of modulation boundary

As $\theta_{B}$ increases, $f$ tends to increase since the overall path length decreases. On the other hand, as $\theta_{B}$ increases, $f$ also tends to decrease since the skyrmion speed drops with larger compensation of the SHE force which comes from a smaller $\delta$ in the case of $\theta_{\text{B}}>\theta_{\text{SkH}}$ (Fig. S2b). Due to the skyrmion size, this compensation also happens for $\theta_{\text{B}}<\theta_{\text{SkH}}$ near the edge and increases with $\theta_{B}$. Therefore, a maximum $f$ should appear due to the competition between these two tendencies. Figure S3 confirms this prediction by plotting $f$ with respect to $\theta_{B}$ for three different values of $\theta_{\mathrm{SkH}}$. The figure and various other results showed that the maximum value of $f$ appears near $\theta_{B}\cong$ 45° and $f$ varies slowly near its maximum value. This is the reason $\theta_{B}$ was chosen as 45° for simplicity in the main text.

**
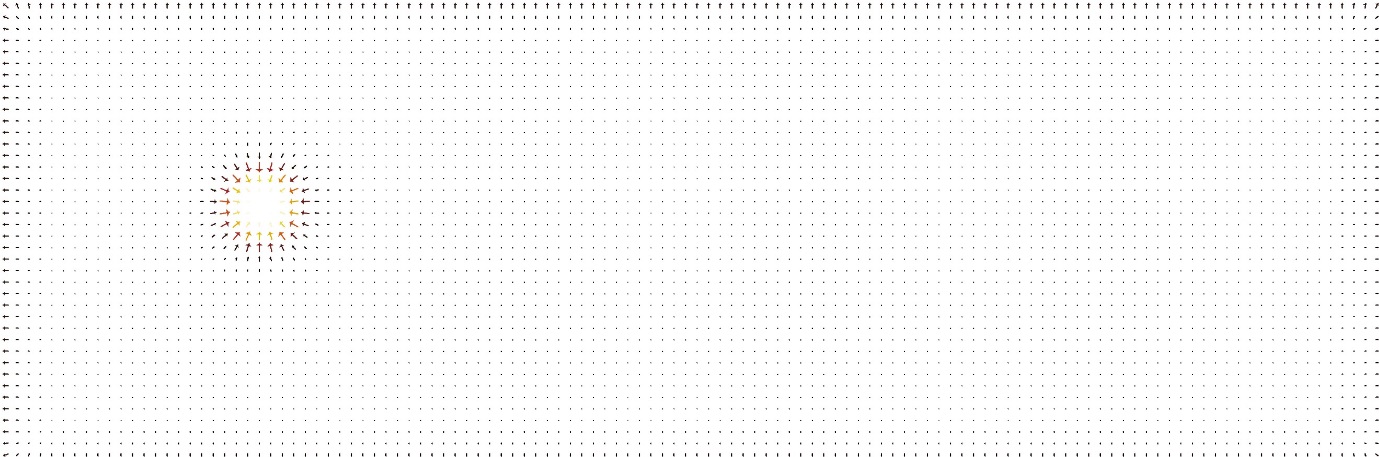
**

**Figure S1 | Initial State of a skyrmion.** Initial state of a Nèel-type up skyrmion with counter clockwise chirality. The in-plane magnetization is indicated with the arrow (length and direction), and the out-of-plane magnetization is indicated with the color (white-up, black-down).


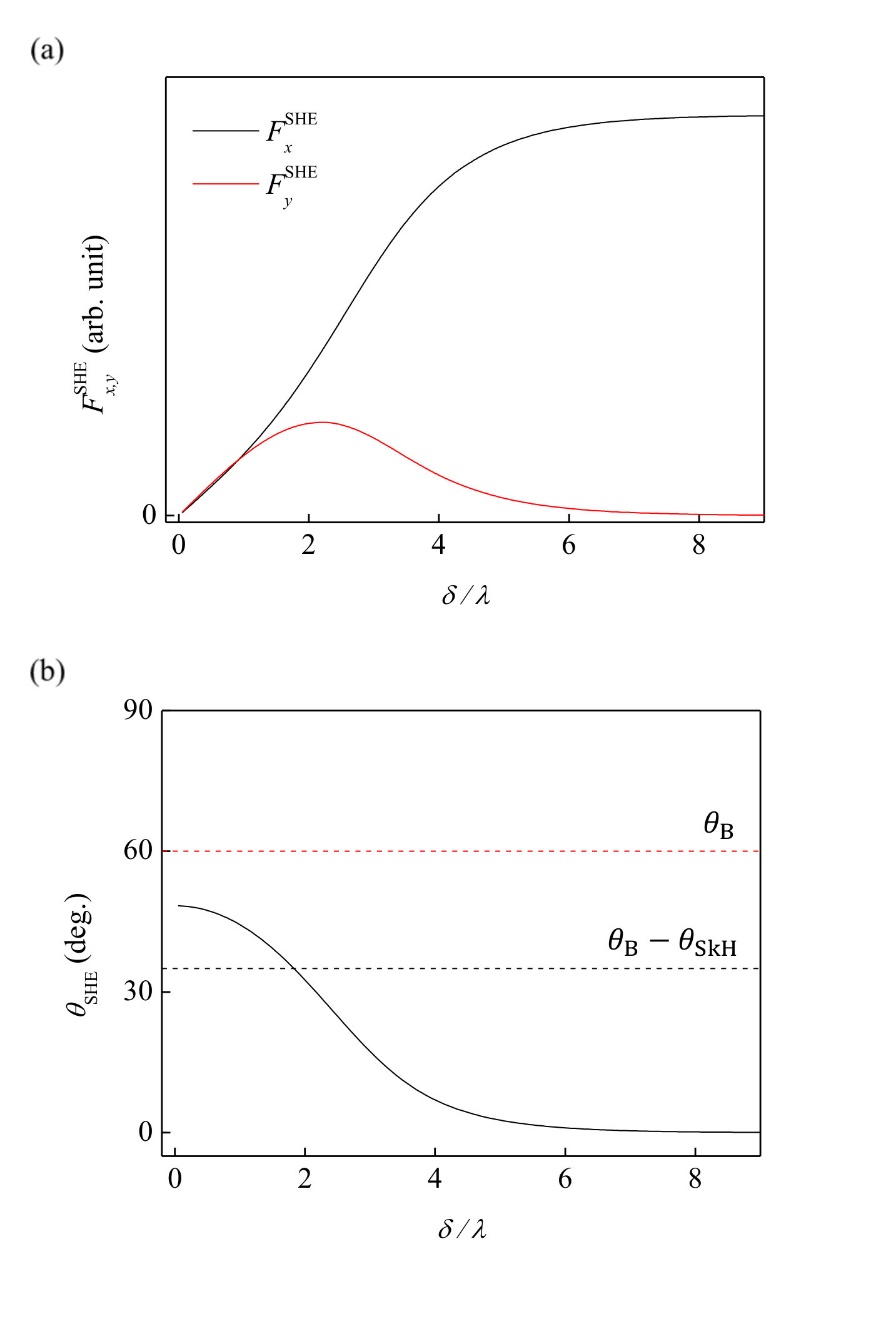


**Figure S2 | SHE force compensated at modulation boundary. a**, Plot of $F_{x,y}^{\mathrm{SHE}}$ with respect to $\delta/\lambda$. **b**, Plot of $\theta_{\mathrm{SHE}}$ with respect to $\delta/\lambda$. The red and black dashed lines show the values of $\theta_{B}$ and $\theta_{B}-\theta_{\mathrm{SkH}}$.


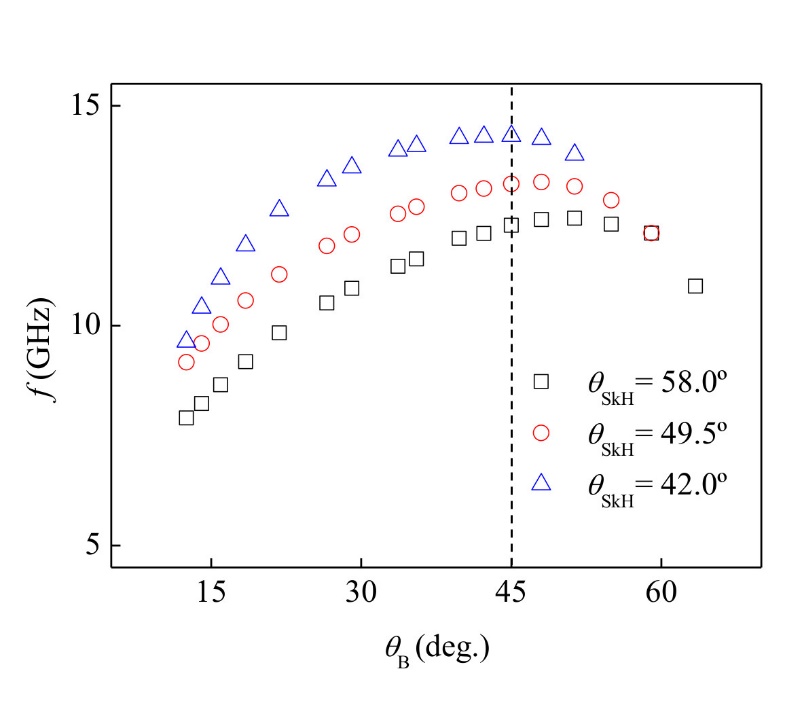


**Figure S3 | Frequency variation with respect to angle of modulation boundary.** Plot of $f$ with respect to $\theta_{B}$ for $\theta_{\text{SkH}}=$58.0, 49.5, and 42.0°, respectively.
